# Supplementary material for: ∆133p53 isoform promotes tumour invasion and metastasis via interleukin-6 activation of JAK-STAT and RhoA-ROCK signalling
Source: Nat Commun. 2018 Jan 17;9:254. doi: 10.1038/s41467-017-02408-0 (PMC5772473; doi:10.1038/s41467-017-02408-0)
Supplement: Supplementary file 1 — Supplementary Information [file 41467_2017_2408_MOESM1_ESM.pdf]

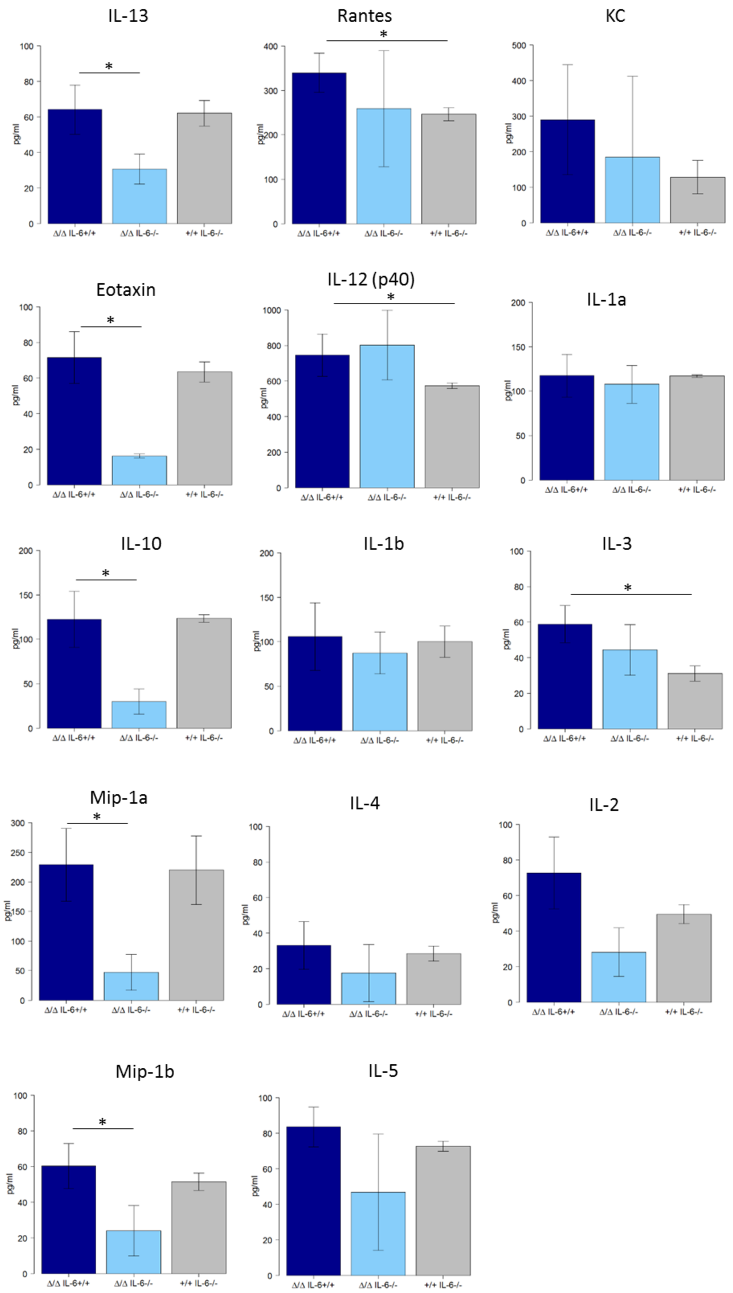

Supplementary Figure 1. Reduced expression of pro-inflammatory cytokines in *IL-6* deficient Δ122p53 mice. Serum from *Δ122/Δ122 IL-6+/+* (n=3), *Δ122/Δ122 IL-6-/-* (n=3) and *+/+ IL-6-/-* (*wtp53 IL-6-/-*)(n=3) mice was analysed using Bio-Plex Pro Mouse Cytokine 23-plex Array. Cytokine concentrations (pg/ml) are means +/- s.e.m. Heteroscedastic one tailed students t.test was performed and $P < 0.05$ was considered significant. $p < 0.05$ - *.

a

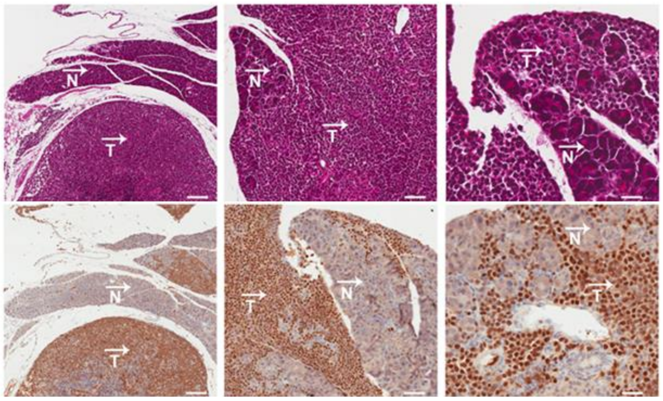

b

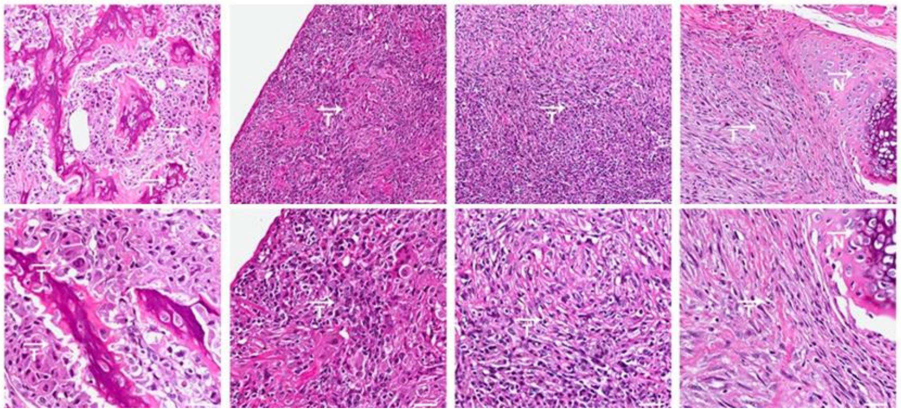

Supplementary Figure 2: Examples of immune staining of lymphomas from *Δ122p53* mice. a. Primary B cell lymphoma from a *Δ122/+ IL-6+/-* mouse. From left to right, top to bottom: pancreatic node, pancreas, pancreas (20x); PAX5 (brown) staining of pancreatic node (4x); PAX5 staining of pancreas; and PAX5 staining of pancreas (20x). From left to right, top to bottom: Scale bar = 125µm, 50µm and 25µm. T denotes tumour and N denotes normal tissue. **b.** Metastatic osteosarcoma from a *Δ122/+ IL-6+/-* mouse. From left to right, top to bottom: primary osteosarcoma in the jaw; metastases (fibrous variant) to spleen; mesenteric node; tail; primary osteosarcoma in the jaw (20x), and metastases to spleen (20x); mesenteric node (20x); and tail (20x). Scale bar = 50µm (top row), scale bar = 25µm (bottom row). 10x magnification unless otherwise stated.

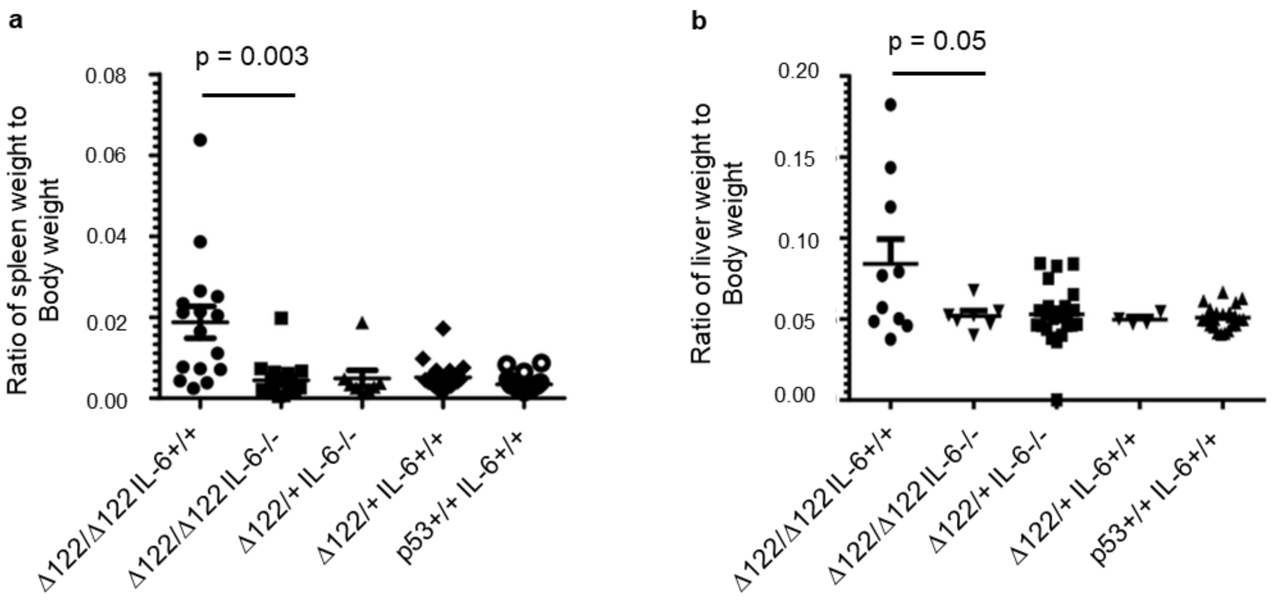

Supplementary Figure 3. IL-6 loss reduces spleen and liver mass in Δ122p53 mice. A. Spleens and livers for all animals that underwent necropsy at time of death were weighed and are expressed as a ratio of spleen weight to total body weight. **a.** spleen weights; **b.** liver weights. Two-tailed unpaired students *t*-test was performed and $p \leq 0.05$ was considered significant.

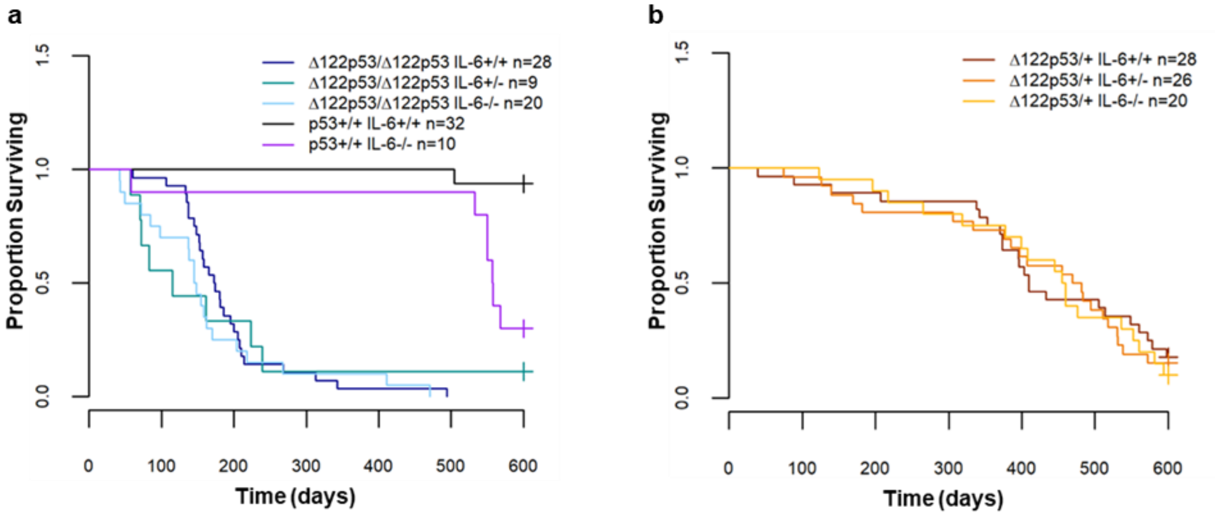

Supplementary Figure 4. Loss of IL-6 in Δ122p53 does not affect life span. A and **B.** Kaplan Meier survival curves for *Δ122/Δ122*, *Δ122/*+ and +/+ mice with varying IL-6 status. All cohorts of mice were followed for 600 days and mice were culled when signs of disease or distressed were observed. **a.** The median survival of *Δ122/Δ122 IL-6*+/+ mice was 173 days, which was reduced to 115 days for mice lacking one IL-6 allele and 148 days for mice lacking both, but neither was significantly different from *Δ122/Δ122 IL-6*+/+ mice (coxph p value = 0.515). +/+ *IL-6* mice had a median survival of 600 days whereas the +/+ mice lacking both alleles of *IL-6* had a median survival of 557.5 days. **b.** *Δ122/*+ mice had a median survival of 409 days, which increased to 475.5 and 457.5 days, with loss of one or two IL-6 alleles, which again was not significant (coxph p value = 0.383). +/+ *IL-6* mice had a median survival of 600 days whereas the +/+ mice lacking both alleles of IL-6 had a median survival of 563 days.

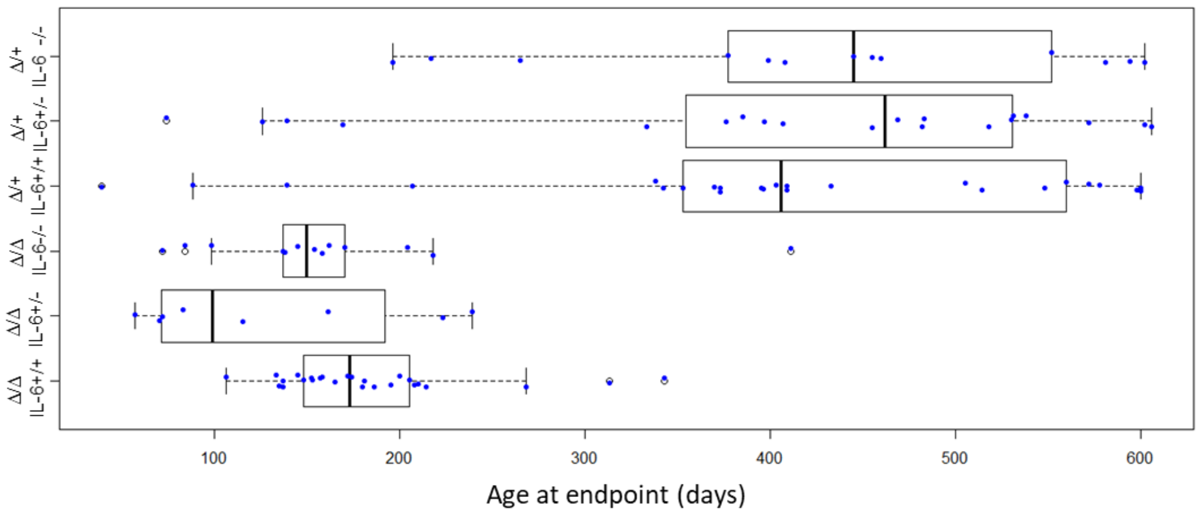

Supplementary Figure 5. Tumour incidence at endpoint. Shows the frequency of tumour incidence by genotype as a boxplot. The line in the middle of each box represents the median, the top and bottom outlines of the box represent the first and third quartiles.

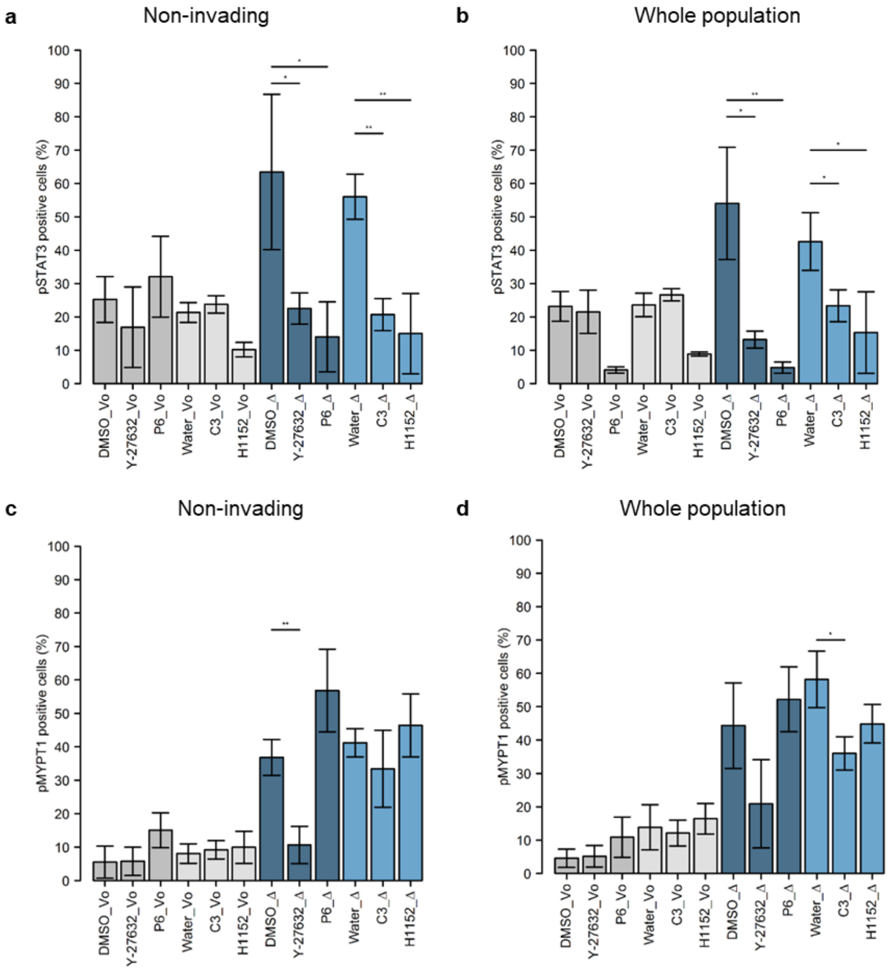

Supplementary Figure 6: Quantitation of pSTAT3 and pMYPT1 in non-invading PDAC/Δ122p53 cells. a. Quantitation of pSTAT3 levels in the non-invading population of PDAC/vector and PDAC/Δ122 cells treated with either a control, the RhoA inhibitor TAT-C3 or the ROCK inhibitor H1152 and stained for expression of pSTAT3. **b.** Quantitation of pSTAT3 levels of the whole population of PDAC/vector and PDAC/Δ122 cells in organotypic matrices treated with either a control, or the above inhibitors. **c.** Quantitation of pMYPT1 levels in the non-invading population of PDAC/vector and PDAC/Δ122 cells treated with either a control, or the various inhibitors. **d.** Quantitation of pMYPT1 levels in the whole population of PDAC/vector and PDAC/Δ122 cells in organotypic matrices treated and quantitated as above. The bars represent the mean and error bars represent ± s.e.m of n=3 repeats with 3 biological replicates each. p values were determined using unpaired t-tests. $p < 0.05$ was considered significant. $p < 0.05$ - *, < 0.005 - ** and < 0.0005 - *** are designated respectively.

Supplementary Table 1: Tumour incidence at endpoint

Comparison	Genotype	<Median	>Median	Chi-square statistic	p - value
Group 1	*Δ/Δ IL-6 +/+*	13	13	1.55	0.213
Group 2	*Δ/Δ IL-6 +/-*	6	2		
Group 1	*Δ/Δ IL-6 +/+*	13	13	3.095	0.078
Group 2	*Δ/Δ IL-6 -/-*	11	3		
Group 1	*Δ/+ IL-6 +/+*	13	13	0.456	0.499
Group 2	*Δ/+ IL-6 +/-*	8	12		
Group 1	*Δ/+ IL-6 +/+*	13	13	0.464	0.495
Group 2	*Δ/+ IL-6 -/-*	5	8		

Summary of chi-square statistic and p value for tumour incidence between the genotypes. $p < 0.05$ was considered significant.

Supplementary Table 2: Summary of Observed / expected Gender Frequency in each genotype.

GENOTYPE	Number of mice	MALE (Observed / Expected)	FEMALE (Observed / Expected)
Δ122/+ IL6+/+	82	39/41	43/41
Δ122/+ IL6+/-	40	29/20	11/20
Δ122/+ IL6-/-	21	8/10.5	13/10.5
Δ122/Δ122 IL6+/+	40	33/20	7/20
Δ122/Δ122 IL6+/-	8	6/4	2/4
Δ122/Δ122 IL6-/-	19	14/9.5	5/9.5

Supplementary Table 3: Summary of colorectal cancer patient clinical data

**Clinical Characteristics**	
Time of tissue collection	1996 - 2007
Median (range) follow up	101.3 (2.2-193.7) months
**Gender**	
Male	17
Female	18
**Grade**	
Well differentiated	2
Moderately differentiated	31
Poorly differentiated	2
**AJCC Stage**	
Stage I	3
Stage II	28
Stage III	1
Stage IV	3
**Recurrence**	
No Recurrence	24
Recurrence	8
Not included	3 (Stage IV patients)
