# Supplementary material for: ∆133p53 isoform promotes tumour invasion and metastasis via interleukin-6 activation of JAK-STAT and RhoA-ROCK signalling
Source: Nat Commun. 2018 Jan 17;9:254. doi: 10.1038/s41467-017-02408-0 (PMC5772473; doi:10.1038/s41467-017-02408-0)
Supplement: Supplementary file 3 — Description of Additional Supplementary Files [file 41467_2017_2408_MOESM3_ESM.pdf]

Description of Additional Supplementary Files

File Name: Supplementary Data 1

Description: Results for expression of transcripts determined by Affymetrix Human Exon arrays positively associated with Δ133TP53 expression determined by RT-qPCR.
