# Supplementary material for: ∆133p53 isoform promotes tumour invasion and metastasis via interleukin-6 activation of JAK-STAT and RhoA-ROCK signalling
Source: Nat Commun. 2018 Jan 17;9:254. doi: 10.1038/s41467-017-02408-0 (PMC5772473; doi:10.1038/s41467-017-02408-0)
Supplement: Supplementary file 4 — Supplementary Data 1 [file 41467_2017_2408_MOESM4_ESM.docx]

**Results for expression of transcripts determined by Affymetrix Human Exon arrays positively associated with ∆*133TP53* expression determined by RT-qPCR.**

| Positively associated with *∆133TP53* | | |
| --- | --- | --- |
| Official Gene Symbol | Transcript Probeset (Core annotations) | Spearman's Correlation |
| *LCK* | 2328843 | 0.58 |
| *MTL1* | 3662249 | 0.56 |
| *GPR114* | 3662776 | 0.55 |
| *IGLL1* | 3954747 | 0.53 |
| *GZMH* | 3558360 | 0.53 |
| *GNAI2* | 2622681 | 0.52 |
| *MT1B* | 3662191 | 0.52 |
| *PPAN* | 3820373 | 0.51 |
| *OR7D4* | 3849490 | 0.50 |
| *OTUD6A* | 3980445 | 0.50 |
| *TMEM169* | 2526972 | 0.50 |
| *KCNIP3* | 2493996 | 0.48 |
| *MT1DP* | 3662171 | 0.48 |
| *EPM2A* | 2977964 | 0.47 |
| *STATH* | 2730178 | 0.47 |
| *GAPT* | 2810765 | 0.47 |
| *MT1P2* | 2462593 | 0.47 |
| *CRNN* | 2435691 | 0.47 |
| *RAMP1* | 2534515 | 0.47 |
| *GBP6* | 2345818 | 0.46 |
| *C1orf211* | 2318216 | 0.46 |
| *SLC6A11* | 2610561 | 0.46 |
| *MT1X* | 3662237 | 0.46 |
| *MT1P1* | 3662113 | 0.46 |
| *PTPRU* | 2327818 | 0.45 |
| *TAAR1* | 2974577 | 0.45 |
| *MT1A* | 3662134 | 0.45 |
| *NAPSA* | 3868424 | 0.45 |
| *XAF1* | 3708076 | 0.45 |
| *SH2D3C* | 3226044 | 0.45 |
| *HMHA1* | 3815495 | 0.45 |
| *MT1F* | 3662203 | 0.45 |
| *LYG1* | 2566712 | 0.45 |
| *CPXCR1* | 3983398 | 0.44 |
| *SCRG1* | 2794007 | 0.44 |
| *GIMAP6* | 3079105 | 0.44 |
| *OR7G1* | 3849465 | 0.44 |
| *OR2T2* | 4045963 | 0.44 |
| *GNRHR2* | 2432608 | 0.44 |
| *ZBED2* | 2688511 | 0.44 |
| *CORO1A* | 3655988 | 0.44 |
| *KCNQ5* | 2913278 | 0.43 |
| *BATF* | 3544611 | 0.43 |
| *NPR1* | 2359784 | 0.43 |
| *ALDH8A1* | 2975259 | 0.43 |
| *C1orf65* | 2382060 | 0.43 |
| *SIRPB1* | 3894728 | 0.43 |
| *OR2F2* | 3029435 | 0.43 |
| *C2orf57* | 2531985 | 0.43 |
| *PYHIN1* | 2362364 | 0.43 |
| *FIBCD1* | 3227483 | 0.43 |
| *PGBD2* | 2390521 | 0.43 |
| *GSTZ1* | 3545404 | 0.42 |
| *KBTBD5* | 2619447 | 0.42 |
| *AK7* | 3550345 | 0.42 |
| *XCL2* | 2443024 | 0.42 |
| *EGFL7* | 3194525 | 0.41 |
| *TRGC2* | 3046521 | 0.41 |
| *DPF1* | 3861245 | 0.41 |
| *SLC45A2* | 2852713 | 0.41 |
| *TMPRSS11A* | 2771878 | 0.41 |
| *JAKMIP1* | 2759159 | 0.41 |
| *GLRX5* | 3550095 | 0.41 |
| *LYL1* | 3852025 | 0.41 |
| *OR8B12* | 3395992 | 0.41 |
| *PPM1D* | 3729538 | 0.41 |
| *CT47B1* | 4020196 | 0.41 |
| *DNALI1* | 2330729 | 0.41 |
| *ACBD7* | 3279080 | 0.41 |
| *PLA2G2D* | 2400060 | 0.41 |
| *KRTAP10-6* | 3934597 | 0.41 |
| *EIF5A* | 3708424 | 0.41 |
| *LETM2* | 3094630 | 0.40 |
| *OSTN* | 2657971 | 0.40 |
| *SREBF1* | 3747971 | 0.40 |
| *CPNE5* | 2952001 | 0.40 |
| *EPHA6* | 2632849 | 0.40 |
| *MAP6D1* | 2708204 | 0.40 |
| *PLCG2* | 3670955 | 0.40 |
| *PTGDS* | 3195041 | 0.40 |
| *PYROXD2* | 3302755 | 0.40 |
| *ATF7* | 3456307 | 0.40 |
| *C22orf40* | 3963975 | 0.40 |
| *CFHR5* | 2373512 | 0.40 |
| *TMPRSS5* | 3391735 | 0.40 |
| *C14orf139* | 3578070 | 0.40 |
| *POTEG* | 3951303 | 0.40 |
| *TGM4* | 2620349 | 0.40 |
| *ZNF429* | 3826695 | 0.40 |
| *AADAC* | 2648078 | 0.40 |
| *ADRB3* | 4049849 | 0.40 |
| *NKG7* | 3868999 | 0.40 |
| *ZFP2* | 2843856 | 0.40 |
| *LRP2BP* | 2796849 | 0.40 |
| *SCO1* | 3745506 | 0.40 |
| *C17orf56* | 3773704 | 0.39 |
| *CTXN1* | 3848645 | 0.39 |
| *MT1E* | 3662140 | 0.39 |
| *NCRNA00242* | 2986150 | 0.39 |
| *KIR2DL2* | 3841839 | 0.39 |
| *RASAL3* | 3853455 | 0.39 |
| *EOMES* | 2667045 | 0.39 |
| *IP6K3* | 2950824 | 0.39 |
| *SLC9A5* | 3665375 | 0.39 |
| *IL31RA* | 2810059 | 0.39 |
| *MRPL49* | 3334920 | 0.39 |
| *GPR87* | 2701055 | 0.39 |
| *IGSF10* | 2701126 | 0.39 |
| *NXPH4* | 3417989 | 0.39 |
| *PRDM8* | 2733291 | 0.39 |
| *ZNF501* | 2620225 | 0.39 |
| *BTN3A1* | 2899373 | 0.39 |
| *SNX3* | 2968245 | 0.39 |
| *SOX13* | 2375917 | 0.39 |
| *LRRN4* | 3896596 | 0.39 |
| *SLC17A3* | 2946113 | 0.39 |
| *C17orf81* | 3708368 | 0.39 |
| *FCRL3* | 2439003 | 0.39 |
| *LAMC3* | 3191878 | 0.39 |
| *NRN1L* | 3665938 | 0.39 |
| *TMEM107* | 3744233 | 0.39 |
| *CFD* | 3815245 | 0.39 |
| *MT3* | 3662097 | 0.39 |
| *PEX11G* | 3848409 | 0.39 |
| *SCRT1* | 4049567 | 0.39 |
| *STK32C* | 3314163 | 0.39 |
| *VN1R5* | 2389998 | 0.39 |
| *C15orf33* | 3623474 | 0.38 |
| *CCDC57* | 3774715 | 0.38 |
| *DHX58* | 3757603 | 0.38 |
| *SLC36A2* | 2881951 | 0.38 |
| *ULBP3* | 2979276 | 0.38 |
| *ZNF454* | 2843885 | 0.38 |
| *CHST2* | 2646127 | 0.38 |
| *RND2* | 3722360 | 0.38 |
| *NLGN1* | 2652806 | 0.38 |
| *SLC23A3* | 2599870 | 0.38 |
| *TRNAU1AP* | 2327544 | 0.38 |
| *C14orf45* | 3544006 | 0.38 |
| *CCL5* | 3753861 | 0.38 |
| *CXCL13* | 2732509 | 0.38 |
| *GALNT13* | 2511050 | 0.38 |
| *GUK1* | 2383917 | 0.38 |
| *THAP9* | 2733900 | 0.38 |
| *ZNF79* | 3189838 | 0.38 |
| *C6orf141* | 2909778 | 0.38 |
| *NPY* | 2993137 | 0.38 |
| *ZNF214* | 3361246 | 0.38 |
| *CDHR4* | 2674749 | 0.38 |
| *CTDP1* | 3795314 | 0.38 |
| *GTSF1L* | 3906710 | 0.38 |
| *MT4* | 3662087 | 0.38 |
| *TLL1* | 2750756 | 0.38 |
| *TRAF3IP3* | 2378134 | 0.38 |
| *ANAPC4* | 2721849 | 0.38 |
| *BCHE* | 2703904 | 0.38 |
| *C21orf94* | 3916965 | 0.38 |
| *FBXO44* | 2320375 | 0.38 |
| *FNDC5* | 2405252 | 0.38 |
| *FSD1* | 3817441 | 0.38 |
| *LRRC4* | 3071470 | 0.38 |
| *PRF1* | 3293442 | 0.38 |
| *PTH1R* | 2621034 | 0.38 |
| *C3orf58* | 2646328 | 0.38 |
| *C5orf48* | 2827178 | 0.38 |
| *FGF9* | 3480888 | 0.38 |
| *PELI3* | 3336223 | 0.38 |
| *WDR49* | 2704144 | 0.38 |
| *WNT9A* | 2459363 | 0.38 |
| *FAM71B* | 2883394 | 0.37 |
| *FUT9* | 2917835 | 0.37 |
| *NCRNA00247* | 2734353 | 0.37 |
| *NDST4* | 2782860 | 0.37 |
| *C3orf51* | 2677625 | 0.37 |
| *MYO1F* | 3849046 | 0.37 |
| *OR4C11* | 3373213 | 0.37 |
| *VIT* | 2477208 | 0.37 |
| *ZYG11A* | 2336540 | 0.37 |
| *BMX* | 3969803 | 0.37 |
| *CCKAR* | 2764492 | 0.37 |
| *ELAC1* | 3788271 | 0.37 |
| *MAGEC3* | 3993488 | 0.37 |
| *MAPK10* | 2776674 | 0.37 |
| *PPP2R2B* | 2880070 | 0.37 |
| *RGS13* | 2372818 | 0.37 |
| *SCAND2* | 3605781 | 0.37 |
| *BTN3A2* | 2899334 | 0.37 |
| *C4orf32* | 2739716 | 0.37 |
| *CNR1* | 2963860 | 0.37 |
| *CPEB3* | 3300244 | 0.37 |
| *CTAG1A* | 4027502 | 0.37 |
| *HIST1H2BO* | 2900118 | 0.37 |
| *STX11* | 2929143 | 0.37 |
| *HOXC12* | 3416269 | 0.37 |
| *PLLP* | 3693142 | 0.37 |
| *SH2B2* | 3016638 | 0.37 |
| *THOC4* | 3774334 | 0.37 |
| *TTC29* | 2788605 | 0.37 |
| *C14orf169* | 3543625 | 0.37 |
| *C1orf74* | 2453873 | 0.37 |
| *LCT* | 2577857 | 0.37 |
| *LIPF* | 3256920 | 0.37 |
| *OR6Y1* | 2439346 | 0.37 |
| *TKTL1* | 3996229 | 0.37 |
| *C6orf126* | 2904816 | 0.37 |
| *CSMD1* | 3121758 | 0.37 |
| *FAM64A* | 3707951 | 0.37 |
| *KLKB1* | 2755120 | 0.37 |
| *MT1G* | 3693000 | 0.37 |
| *PYDC1* | 3688325 | 0.37 |
| *C14orf49* | 3578090 | 0.37 |
| *CNGA3* | 2495414 | 0.37 |
| *NEUROD1* | 2590493 | 0.37 |
| *TSHB* | 2353068 | 0.37 |
| *CIDECP* | 2662524 | 0.37 |
| *IFI35* | 3722343 | 0.37 |
| *LIMD2* | 3766271 | 0.37 |
| *MLST8* | 3644598 | 0.37 |
| *PALM* | 3815123 | 0.37 |
| *REC8* | 3529727 | 0.37 |
| *ZMYND12* | 2408930 | 0.37 |
| *ZNF718* | 2713842 | 0.37 |
| *ADSSL1* | 3554361 | 0.37 |
| *C1orf162* | 2351857 | 0.37 |
| *ITPKA* | 3590470 | 0.37 |
| *KCNJ8* | 3446912 | 0.37 |
| *SLC2A6* | 3228728 | 0.37 |
| *TFDP3* | 4022354 | 0.37 |
| *DENND1C* | 3847907 | 0.36 |
| *ITGB7* | 3456050 | 0.36 |
| *PI16* | 2905298 | 0.36 |
| *SLC4A1* | 3759023 | 0.36 |
| *TMEM190* | 3842133 | 0.36 |
| *ZNF691* | 2332862 | 0.36 |
| *ZNF818P* | 3840647 | 0.36 |
| *MLN* | 2950888 | 0.36 |
| *CAMKV* | 2674847 | 0.36 |
| *CR2* | 2377285 | 0.36 |
| *LHFPL5* | 2904837 | 0.36 |
| *MCM9* | 2971702 | 0.36 |
| *PIK3C2G* | 3406885 | 0.36 |
| *RGR* | 3255362 | 0.36 |
| *C1orf187* | 2320394 | 0.36 |
| *C2orf40* | 2498279 | 0.36 |
| *C8orf48* | 3086923 | 0.36 |
| *CEND1* | 3358394 | 0.36 |
| *CYLC2* | 3182686 | 0.36 |
| *C17orf55* | 3773802 | 0.36 |
| *C1orf64* | 2322212 | 0.36 |
| *EPYC* | 3465209 | 0.36 |
| *GATA5* | 3913277 | 0.36 |
| *KISS1R* | 3815269 | 0.36 |
| *NEK10* | 2666808 | 0.36 |
| *PDLIM2* | 3089573 | 0.36 |
| *SLAIN1* | 3494730 | 0.36 |
| *TPO* | 2466564 | 0.36 |
| *CLEC14A* | 3561870 | 0.36 |
| *CSAG1* | 3995332 | 0.36 |
| *FCRLA* | 2363853 | 0.36 |
| *GH2* | 3766513 | 0.36 |
| *OR9H1P* | 2390163 | 0.36 |
| *PFAS* | 3709541 | 0.36 |
| *PRSS50* | 2672299 | 0.36 |
| *RCSD1* | 2365873 | 0.36 |
| *RIMS1* | 2913125 | 0.36 |
| *AMIGO3* | 2674647 | 0.36 |
| *ETV7* | 2951860 | 0.36 |
| *FOXD4* | 2501472 | 0.36 |
| *GRID2* | 2736062 | 0.36 |
| *TYMS* | 3775844 | 0.36 |
| *ZNF114* | 3837711 | 0.36 |
| *KCNK10* | 3575242 | 0.36 |
| *OLFM3* | 2425654 | 0.36 |
| *PCDH9* | 3516642 | 0.36 |
| *PPY2* | 3715477 | 0.36 |
| *SOX18* | 3914288 | 0.36 |
| *ZNF626* | 3856244 | 0.36 |
| *ZNF683* | 2402692 | 0.36 |
| *C4orf45* | 2791594 | 0.36 |
| *EN1* | 2572910 | 0.36 |
| *GPR139* | 3683432 | 0.36 |
| *OR4A16* | 3330588 | 0.36 |
| *OR52N4* | 3318503 | 0.36 |
| *TPPP2* | 3527810 | 0.36 |
| *CD3G* | 3351302 | 0.36 |
| *FRAS1* | 2732660 | 0.36 |
| *PACSIN3* | 3371966 | 0.36 |
| *RAC3* | 3738472 | 0.36 |
| *TRIM25* | 3763664 | 0.36 |
| *TRIM46* | 2360729 | 0.36 |
| *ACCN5* | 2791010 | 0.35 |
| *BATF3* | 2454819 | 0.35 |
| *C10orf54* | 3293726 | 0.35 |
| *DVL2* | 3743441 | 0.35 |
| *LRRC7* | 2341394 | 0.35 |
| *NRXN1* | 2552646 | 0.35 |
| *SLC27A6* | 2827666 | 0.35 |
| *TRIML2* | 2797783 | 0.35 |
| *TUBB2B* | 2939245 | 0.35 |
| *ADAMTSL4* | 2358394 | 0.35 |
| *ADORA3* | 2427983 | 0.35 |
| *KLRG1* | 3404036 | 0.35 |
| *LILRA4* | 3870800 | 0.35 |
| *NGFR* | 3725686 | 0.35 |
| *SIX1* | 3567357 | 0.35 |
| *TSPAN4* | 3316376 | 0.35 |
| *TSPY1* | 4029734 | 0.35 |
| *APITD1* | 2319833 | 0.35 |
| *ATP8B4* | 3623553 | 0.35 |
| *ELP3* | 3091633 | 0.35 |
| *HBZ* | 3642645 | 0.35 |
| *ICAM1* | 3820459 | 0.35 |
| *IFNG* | 3461106 | 0.35 |
| *PON1* | 3061943 | 0.35 |
| *SLC36A3* | 2881924 | 0.35 |
| *VSX1* | 3901771 | 0.35 |
| *C3orf75* | 2672775 | 0.35 |
| *CHST12* | 2987524 | 0.35 |
| *GHRL* | 2662628 | 0.35 |
| *GPR45* | 2497983 | 0.35 |
| *OR9Q1* | 3331681 | 0.35 |
| *RGS8* | 2447196 | 0.35 |
| *SEMA6C* | 2434807 | 0.35 |
| *SPDYE3* | 3015415 | 0.35 |
| *ZNF165* | 2900201 | 0.35 |
| *AGRP* | 3695727 | 0.35 |
| *C1orf49* | 2369254 | 0.35 |
| *CPZ* | 2717859 | 0.35 |
| *KIAA1407* | 2689287 | 0.35 |
| *PNPLA5* | 3963142 | 0.35 |
| *SPN* | 3655576 | 0.35 |
| *TCP10* | 2985130 | 0.35 |
| *CCDC72* | 2621693 | 0.35 |
| *FBXL16* | 3675309 | 0.35 |
| *KCNJ12* | 3714784 | 0.35 |
| *MMEL1* | 2392667 | 0.35 |
| *NRTN* | 3818143 | 0.35 |
| *NTSR1* | 3892874 | 0.35 |
| *RAB40B* | 3775212 | 0.35 |
| *ZNF554* | 3816646 | 0.35 |
| *FEZF2* | 2679378 | 0.35 |
| *GFI1* | 2422972 | 0.35 |
| *GP1BA* | 3707337 | 0.35 |
| *LRRC3* | 3923766 | 0.35 |
| *TAS2R40* | 3028967 | 0.35 |
| *C1orf86* | 2392096 | 0.35 |
| *CAPN14* | 2547091 | 0.35 |
| *DFFB* | 2317514 | 0.35 |
| *GALR2* | 3735384 | 0.35 |
| *IL5RA* | 2660618 | 0.35 |
| *MAMDC4* | 3194835 | 0.35 |
| *MYOC* | 2443953 | 0.35 |
| *TTC18* | 3294362 | 0.35 |
| *ZNF385A* | 3456701 | 0.35 |
| *C3orf20* | 2612014 | 0.35 |
| *HAPLN4* | 3855620 | 0.35 |
| *IL12B* | 2884302 | 0.35 |
| *NECAB1* | 3106480 | 0.35 |
| *TREM2* | 2953502 | 0.35 |
| *WDR69* | 2530735 | 0.35 |
| *CACNG7* | 3841137 | 0.35 |
| *FAM115C* | 3029261 | 0.35 |
| *POMC* | 2544632 | 0.35 |
